# Hospital Expenditure: The Commissariat

**Published:** 1896-03-14

**Authors:** 


### The Institutional Workshop.

### HOSPITAL EXPENDITURE.—THE COMMISSARIAT.

### XI.—THE COST OF THE PATIENT.

It will have become clear to those who have followed the percentages given in the preceding articles that the patients, though numerically by far the strongest element in the hospital household, are not, as is commonly assumed, the main persons to be considered in reckoning the expense of provisions. The subject of domestic economy in hospitals, is too often treated as though the patients' diet were the only point deserving attention, and the public has grown so accustomed to this fiction that they are occasionally betrayed into a feeling of injured surprise at the money spent on the board of the necessary attendants. The percentage of attendants or officials to patients differs widely in different establishments, but the returns of the last census make it possible to ascertain the average. Here we find the total number of inmates in hospitals and infirmaries given as 40,341, of which number only 27,579 were actual patients. The number of officials then is 12,762, or 31 per cent. of the total hospital population. When it is remembered that these 31 per cent. are persons in vigorous health, and engaged for the most part in very anxious and wearing work, spending probably the best years of their life in the institution, it does not take much consideration to see that the cost of boarding each one is likely to exceed that of boarding three or four patients, who, even in convalescence, are entirely inactive, and who seldom remain more than a month under treatment. In small hospitals, then, where the proportion of officials rises to over 40 per cent. of the inmates, the cost of the patients' board will be considerably less than half the total expenditure on provisions, and even in the largest hospitals the board of patients will not cost much more than that all of the attendants.

But if not always the most expensive, the patients certainly constitute by far the most complex part of the hospital household. Three difficulties make themselves felt as soon as any attempt is made to compare the cost of patients in different institutions. There is first the wide difference, which is common to all general hospitals, between the needs of one patient and another, between the cost of low or fever diet, consisting mainly of milk; the cost of full, extra or convalescent diets, consisting of abundance of plain and nourishing food; and thirdly, that of special diets, consisting, in part at any rate, of more expensive delicacies necessary in certain medical cases. What is the cost of each form of diet, and what is the average proportion of patients assigned to each? These questions are rendered more complicated by the second difficulty, which consists in the distinctive character of the patients in many general hospitals. In some the majority of cases are surgical, and when these patients are nearly all labouring men, the cost of maintaining them will not bear fair comparison with that of maintaining wards provided for women and children. The noble work performed at great cost by the Middlesex Hospital, in the maintenance of the cancer wards, is another illustration of the existence of special features in general hospitals, rendering mutual comparison a matter of extreme delicacy. And, lastly, there is the standing crux of difference of administration, which in many hospitals provides patients with every article of diet they require; in others, leaves certain items to be provided by the patient; in others, again, to be supplied when necessary by the Samaritan Fund.

Bearing all these points in mind, it is rather surprising to find that the cost of the patients' diet in some of the London general hospitals, where the accounts for the household are kept entirely distinct, shows an almost identical average one with another.

St. Thomas's, with a daily average of 365 patients, expended in 1893 the sum of £6,148 on the patients' board. The annual cost per head is £16 5s., or a weekly average of 6s. 4½d., including tea, sugar, and butter, and the average of the patients' board reaches 61·7 per cent. of the total amount expended on provisions.

At St. Bartholomew's the cost is identical. A daily average of 536 patients are boarded at a cost of £8,957 a year. The annual cost per head is again £16 5s., including tea, sugar, and butter. The patients' percentage of the total cost of provisions is 62·1.

At Guy's the cost of the patients' board is slightly less; 411 patients cost £6,256 a year. The annual cost is £15 a year, and the weekly average 5s. 9d., again including everything. The patients' percentage of the total cost of provisions is again much the same—61·5.

At Westminster the board of 176 patients costs £2,816 a year. This equals £16 a year each, or about 6s. 1¾d. a week, including everything. The percentage of the total cost of provisions to be assigned to the patients is rather higher than in the preceding hospitals, and amounts to 64·4. As the accounts for patients and household are not rendered separately at the Westminster Hospital, it may be mentioned that this calculation is based on the following deductions for members of the household: Nurses, £900; doctors, £466; servants (at 6s. a week), £187.

The cost of patients can be ascertained in the same way at the Royal Free Hospital by deducting for doctors £245; household, including nurses, £1,466; lunches £93. This leaves an expenditure of £1,608 on the board of a daily average of 112 patients. The annual cost of patients per head is thus £14, or about 5s. 4½d. a week. It must be remembered that this amount does not include, tea, sugar, or butter, except for a certain proportion (estimated at about one-seventh of the whole number) of very poor patients. If the cost of these articles were added the amount spent on the patients' board would be almost identical with that of the other hospitals quoted. The cost of the patients' board is 52·3 per cent. of the whole cost of provisions.

At University the cost of patients' board, minus tea, sugar, and butter, is identical with that at the Royal Free. From the total expended on provisions must be deducted £2,262 for nurses, £600 for doctors, and about £187 for lunches and for board of servants. It will then be found that a daily average of 186 patients are fed at a cost of £2,656 a year. The yearly cost of each patient is again £14. The patients' board amounts to 47·4 per cent. of the total cost of provisions.

It cannot be considered other than satisfactory that the amounts expended on provisions for such widely varying numbers of sick persons in some of the best known London hospitals should be found to tally so accurately within a penny or two a week. It is only matter for great regret that data are not furnished by all hospitals with respect to this important point.

Every effort has been made to ascertain the cost of patients' board in provincial hospitals, but with a very moderate degree of success, owing to the unsatisfactory system, which is almost universal, of keeping but one account of the expenditure on staff, household, and patients.

The Birmingham General Hospital, by an admirable system of which we propose to give some account later on, ascertains the weekly cost for each article of diet of patients and household respectively. By the courtesy of the secretary, we have been favoured with the average for the summer quarter of 1895, and find the weekly cost per head of the in-patients works out at slightly over 4s. a week. This includes also malt liquor, wine, and spirits.

At the Glasgow Western Infirmary the accounts for patients and household are also kept entirely separate, The average cost of patients' board, taking the last three years together, works out at about 5s. 4d. a week, or for 1894-5 at 5s. 2d.

At the Derbyshire Royal Infirmary, where everything is provided for the patients, and where the diet is excellent, the cost of the in-patients has been accurately worked out, and is found to amount to 4s. 1d. per head a week.

